# Exploring the Role of Salt Supplementation on Milk Composition, Fatty Acids, and Insulin Response in Lactating Camels

**DOI:** 10.3390/vetsci12010022

**Published:** 2025-01-06

**Authors:** Riyadh S. Aljumaah, Ahmed A. K. Salama, Mutassim M. Abdelrahman, Moez Ayadi, Gerardo Caja, Mohammed A. Alshaikh, Mohammed A. Al-Badwi, Abdulkareem M. Matar

**Affiliations:** 1 Department of Animal Production, College of Food and Agriculture Sciences, King Saud University, P.O. Box 2460, Riyadh 11451, Saudi Arabia; rjumaah@ksu.edu.sa (R.S.A.); amutassim@ksu.edu.sa (M.M.A.); moez.ayadi@isbb.rnu.tn (M.A.); alshaikh@ksu.edu.sa (M.A.A.); malbadwi@ksu.edu.sa (M.A.A.-B.); 2 Group of Research in Ruminants (G2R), Department of Animal and Food Sciences, Universitat Autònoma de Barcelona, 08193 Bellaterra, Spain; ahmed.salama@uab.cat (A.A.K.S.); gerardo.caja@uab.es (G.C.); 3 Department of Animal Biotechnology, Higher Institute of Biotechnology of Beja, University of Jendouba, Beja 9000, Tunisia

**Keywords:** dromedary camel, dietary salt, milk composition, fatty acids, insulin

## Abstract

Camel milk is a valuable food source with unique nutritional properties and potential health benefits. This study investigated the influence of high dietary salt on milk composition and fatty acid (FA) profile as well as insulin regulation in dairy camels. Twelve multiparous female camels were used in a crossover design with two treatments: control concentrate (CON; 1.3% salt) and high-salt concentrate (SAL; 3.9% salt). Each period lasted 3 weeks, with camels switched between treatments in the second period. The measured variables included milk yield, milk composition, blood metabolites, and insulin levels in blood and milk. The SAL group exhibited higher (*p* < 0.01) water consumption. Nevertheless, milk yield and composition (fat, protein, lactose) remained unaffected. Notably, SAL camels had elevated blood insulin levels (*p* < 0.05) compared to the CON group, suggesting enhanced pancreatic activity possibly driven by osmotic balance changes. Milk FA profiles revealed a reduction in unsaturated fatty acids (UFA, *p* < 0.04), particularly monounsaturated (MUFA, *p* < 0.05) and odd-chain fatty acids (OCFA, *p* < 0.05). Furthermore, lipid quality indices such as the atherogenic index (*p* < 0.01) and the hypocholesterolemic/hypercholesterolemic FA ratio (h/H, *p* < 0.01) indicated a less favorable milk fat profile in the SAL group. These findings suggest that while moderate salt supplementation may not negatively impact milk yield in dairy camels, it alters both metabolic and milk fat composition variables, with potential implications for the nutritional quality of milk.

## Simple Summary

This study looked at how adding extra salt to the diet of dairy camels affects their milk and overall metabolism. The camels that consumed more salt drank more water and had higher levels of insulin in their blood. However, the milk from the salt-fed camels contained lower levels of healthy fats, specifically unsaturated fats, which are important for heart health. These results suggest that adding a high amount of salt to the diet may affect the quality of milk and potentially make it less healthy due to the shift in fat types. In simple terms, a moderate increase in dietary salt for dairy camels changes the type of fat in their milk and increases blood insulin levels, which could affect the health benefits of the milk and the camels’ insulin metabolism.

## 1. Introduction

Many countries around the world, especially in the Middle East, are working to promote the importance of camel milk and consider it a valuable alternative, primarily produced by Bedouins. As desert animals, camels have developed several adaptations to survive in harsh environments characterized by limited water, poor-quality feed, and high temperatures. They are large, strong, durable, and extremely resilient [1]. Although camels are not bred specifically for milk production, they can continue producing milk for 18 months, even under extreme environmental conditions. Therefore, they are vital to the inhabitants of dry areas, serving as both a means of transportation and a source of food. Despite their lower milk production compared to cows, camels rank as the fifth-largest milk producers in the world [2].

Camel milk has been reported to possess antidiabetic properties, potentially due to its high levels of insulin and insulin-like proteins as well as bioactive peptides that may regulate blood glucose levels [3]. Studies have shown that patients with type 2 diabetes can reduce their fasting blood glucose levels and increase their insulin sensitivity by regularly consuming camel milk [4,5]. Additionally, camel milk has anti-inflammatory and antioxidant properties that may help prevent complications from diabetes [6]. However, research specifically examining the influence of dietary salt on fatty acid profile and insulin levels in the blood and milk of ruminants remains limited [7], and to our knowledge, there are no studies that have examined this relationship in camels.

Several factors, including diet composition, breed, and management system, may affect the levels of insulin and insulin-like peptides in camel milk. Among dietary factors, salt intake may influence blood insulin levels and, consequently, the insulin content in milk. Evidence from human and animal models suggests a link between salt consumption and insulin dynamics, where increased Na^+^ intake can lead to hyperinsulinemia, likely due to Na^+^ effects on cellular ion channels and pancreatic function [8,9]. High salt consumption may also increase tight junctions’ permeability in the mammary gland, potentially allowing more insulin to pass from blood to milk. Although direct studies on the mammary gland are scarce, a recent study in rats demonstrated that high Na^+^ intake can disrupt gut tight junctions’ integrity and increase permeability [10].

While mineral salts have been studied extensively in dairy cows [11] and sheep [12], and the effect of salt on fatty acids (FA) has been reported, several studies have found that the addition of mineral salts, particularly sodium bicarbonate, increases rumen pH. This increase in pH can improve rumen fermentation by favoring the production of acetate over propionate [13]. This shift in hydrogenated fatty acid production could help alleviate the problems of low-fat milk in dairy cows [14]. In addition, sodium bicarbonate and other mineral salts increase the rate of dilution of rumen fluid. This increased dilution rate may also contribute to an increase in the acetate-propionate ratio, particularly in cattle fed high-concentration feed [13]. However, there is a significant research gap regarding their effects on camel milk composition, particularly in relation to FA profiles, since the FA profile of camel milk plays a significant role in its nutritional value and potential health benefits for humans. The greater content of unsaturated FA (UFA) and the larger casein micelles in camel milk result in a softer clot in the stomach, allowing for faster and more efficient digestion compared to cow’s milk. Additionally, the smaller fat globules in camel milk further support digestion [15]. These characteristics make camel milk more suitable for individuals with bovine milk intolerance [16]. Due to its nutritional value and numerous health benefits, camel milk is often referred to as the “white gold of the desert” [17]. While marketing in Saudi Arabia is primarily done locally, camel milk is gradually gaining recognition in the international market.

To address the above-mentioned research gaps, we hypothesized that a moderate increase in dietary salt levels in dairy camels would alter systemic insulin dynamics, which might increase milk insulin content and modify the FA profile. This study is the first to comprehensively analyze the effects of increased dietary salt levels on both blood and milk insulin levels in dairy camels, as well as key FA related to human health, particularly those influencing cardiovascular disease risk. The objectives of the current study were to evaluate the impacts of two levels of dietary salt on milk yield, milk composition, and blood metabolites in dairy camels, with a specific focus on blood and milk insulin levels and the FA profile of milk.

## 2. Materials and Methods

### 2.1. Animals, Treatments, and Diets

Twelve multiparous female camels (578 ± 24 kg average body weight; 3.1 ± 0.3 parities; 105 ± 22 days in milk) were used in a crossover design with two periods, each lasting 3 weeks. In the first period, 6 camels were fed a control concentrate (CON; 1.4% salt; 99.7% purity), while the remaining 6 were fed a high-salt concentrate (SAL; 4.3% salt; 99.7% purity). In the second period, the groups were switched, with the CON camels from the 1st period receiving SAL, and the SAL camels receiving CON. The ingredients of CON and SAL concentrates are shown in Table 1.

Animals were individually fed 6.5 kg of experimental concentrates (CON or SAL) and 3.8 kg of alfalfa hay daily. All animals consumed the full amounts of concentrates and alfalfa hay offered. Fresh water was provided separately to each treatment group early in the morning. Daily water consumption was recorded using a 200-L water trough, which was filled each morning. After the evening milking, the remaining water was measured, and the trough was emptied.

All animals were kept under the same management conditions in the spring, from February to April 2023. Camels were hand-milked twice daily (9 a.m. and 5 p.m.). Calves stayed with their mothers from after the evening milking until 5 a.m.

### 2.2. Samples, Measurements, and Chemical Analysis

Daily records of milk production were maintained for the last seven days of each period. Milk samples (120 mL) for composition were taken during days 6 and 7 of each period. These samples were immediately placed in an ice chest and transported to the biotechnology laboratory of the Animal Production Department. For the FA profile, milk samples from both days were combined, and a representative sample was taken. Feed samples were also collected and stored at 4 °C until the chemical analysis.

The dry matter, protein, ether extract, NDF, ADF, and ash contents of feeds (Table 2) were determined according to the official methods of the AOAC [18]. Fat was extracted from feed samples using Soxhlet extraction (Soxtec 8000 extraction device-FOSS-Nils Foss Allé 1-DK-3400 Hilleroed-Denmark). The extracted fat was analyzed in triplicate to determine the FA profile of the feeds (Table 2).

Infrared technology was used with the Milkoscan™FT+ device (Foss Electric-Allé 1-DK-3400 Hilleroed, Denmark) to measure the concentrations of fat, lactose, protein, total solids (TS), and solids non-fat (SNF) in milk. Insulin was determined in skimmed milk using a bovine ELISA kit (Mercodia Diagnostics, Uppsala, Sweden) according to the manufacturer’s protocol. Reacted ELISA plates were read using an automatic reader (RX Monza, Randox, UK) at 450 nm.

Milk samples were centrifuged for 15 min at 4 °C and 3500 rpm to separate the fat. The top fat layer was then collected and stored in glass tubes at −20 °C until further analysis [19]. For the methylation of FA, 1 mg of milk fat was placed in glass tubes, followed by the addition of 500 µL of hexane (99%) and 250 µL of sodium methoxide (0.5 M in methanol) to prevent the isomerization of UFA. The mixture was vortexed for 10 s. The tubes were then placed in a beaker of distilled water at 50 °C for 20 s, removed, and cooled to 24 °C for 2 min. Then, 250 µL of 5% methanolic hydrochloric acid was added to the cooled samples to extract all fat, and the samples were vortexed again for 10 s. The tubes were returned to the beaker at 50 °C for an additional 20 s. After cooling, 1000 µL of hexane was added to dissolve and extract pure fat. Finally, the hexane layer was carefully removed from the top of the tube and transferred into vials, which were cooled at 4 °C for subsequent chromatographic analysis.

The FA methyl esters were analyzed using a gas chromatography-mass spectrometer (GC-MS). Helium (1.8 mL/min) was used as the carrier gas, with injector and detector temperatures set at 245 °C. The oven temperature was initially set at 60 °C (1 min) and then increased to 120 °C at a rate of 20 °C/min, maintaining this final temperature for 15 min [20]. Samples were injected at a ratio of 1:20 into a BPX-70 capillary column (60 m × 0.25 mm × 0.25 mm; SGE, Melbourne, Australia). The FAs were identified by comparing the retention time of the peaks with those obtained by certified standards (FAME MIX C6-C24, CLA FAME, Sigma Aldrich, St. Louis, MO, USA). Peaks were integrated using data acquisition software (Agilent ChemStation, version B.04.01), and FA quantification was performed using an external standard calibration. The results were expressed in grams per 100 g of fat.

At the end of each period, blood samples were collected, and plasma was separated and frozen for the analysis of insulin and other metabolites. Insulin concentrations in blood plasma were determined using a bovine ELISA kit (Mercodia Diagnostics) and reacted ELISA plates were read by an automatic reader at 450 nm. Quimica Clinica Aplicada kits (QCA, Tarragona, Spain) were used to determine the concentrations of glucose, total protein, albumin, globulin, triglycerides, and urea. Analyses were performed using colorimetric methods with the RX Monza chemical analyzer spectrophotometer (Randox Laboratories Ltd., Crumlin, UK).

### 2.3. Lipid Quality Indices

The values of saturated FA (SFA), MUFA, and polyunsaturated FA (PUFA) were used to calculate lipid quality indices as follows:

Thrombogenic index (TI) =$\;\frac{C12:0+C16:0+C18:0}{\left(0.5\times MUFA\right)+\left(0.5\times n-6\right)+\left(3\times n-3\right)+\left(n-3:n-6\right)}$ [21].

Atherogenic index (AI): $\frac{\left(C12:0)+(4\times C14:0)+(C16:0\right)}{\left(MUFA+PUFA\right)}$

Hypocholesterolemic fatty acids (hFA) =C18:1+PUFA;

Hypercholesterolemic fatty acids (HFA) = C12:0 + C14:0 + C16:0;

hFA:HFA ratio (h/H) $=\frac{hypocholesterolemia\;ratio}{hypercholesterolemic\;ratio}$ according to Santos-Silva et al. [22].

The transfer rates of OA, LA, and ALA were calculated using their intake and milk output values according to Stergiadis et al. [23] as follows:

FA content in milk (g)/FA intake (g), where:

$$\begin{array}{l} \left(FA\;content\;in\;milk\;(g\right)\\ =milk\;yield\;(g)\times [milk\;fat\;content\;(g/kg\;milk)\;/\;1000)]\times [FA\;(g\\ /kg\;total\;FA)\times 0.933\\ /1000)],\;with\;0.933\;representing\;\%\;of\;FA\;in\;total\;milk\;fat) \end{array}$$

$$\begin{array}{ll} \left(FA\;intake\;(g/d\right) & \\ & =DMI\;(g)\times [feed\;lipid\;content\;(g/kg\;DM)\;/\;1000]\\ & \times [FA\;(g/kg\;total\;FA)\;in\;feed\;/\;1000]). \end{array}$$

### 2.4. Statistical Analyses

The MIXED procedure of SAS version 9.4 (SAS Institute, Inc., Cary, NC, USA) was used to analyze the repeatedly measured data (i.e., water intake, milk yield, and milk composition). The statistical mixed model considered the effects of treatment (CON vs. SAL) and period as fixed effects, the interaction of treatment × period, the random effect of the animal, and the residual error. Since the experimental design was a crossover with two experimental periods, the interaction treatment × period accounted for the possible residual treatment effect from the previous period.

For the data measured only once at the end of each period (i.e., milk FA and blood metabolites), a GLM procedure was used. The model included the effects of treatment, period, and their interaction. The normality of the data distribution was checked, and transformations were applied to blood insulin values. The Tukey test was used to identify differences in least square means. Significance was declared at *p* < 0.05 and tendencies at *p* < 0.10. The XLstat-2021 was used to calculate the principal component analysis (PCA).

## 3. Results

### 3.1. Performance and Milk Production

Camels on the SAL diet showed a 35% reduction in feed intake of the SAL concentrate mixture during the initial phase of the adaptation period. However, as they acclimated, their intake of the concentrate mixture increased, and they fully consumed the SAL concentrate by the end of the adaptation period. During the 7 days of measurements during each period, camels in two groups consumed the whole amounts of concentrate mixture and alfalfa hay. Additionally, SAL camels consumed 5.3% more water overall compared to the CON animals. Salt treatment had no effect on body weight (593 ± 26 kg, on average; *p* = 0.87), but body weight increased numerically (*p* = 0.56) from 585 ± 23 kg at the end of period 1 to 601 ± 24 kg at the end of period 2.

The addition of salt had no effect on milk yield (4.4 ± 0.4 kg/d, on average), milk composition (fat, protein, and total solids), or milk freezing point (Table 3). Furthermore, milk insulin levels did not vary between the CON and SAL groups. No significant effects of period or period × treatment interaction (*p* > 0.20) were detected for milk yield or any milk components.

### 3.2. Milk Fatty Acid Profile

Table 4 presents the milk fatty acid (FA) profile of camels fed the CON and SAL concentrates. The addition of salt increased (*p* < 0.05) the content of short-chain FA (SCFA: C6:0 and C8:0) and medium-chain FA (MCFA: C14:0 and C16:0). In contrast, camels fed the SAL diet had lower (*p* < 0.05) levels of most odd-chain FA (OCFA: C15:0 iso, C15:0 antiso, C17:0 antiso, and C17:1 cis10), C16:0 iso, C18:1 trans7, C18:1 trans11, C18:1, and C18:2 trans9,12 compared to the CON group. Additionally, C16:1 cis9 tended to be lower (*p* < 0.10) in the SAL group.

The total milk FA profile as well as lipid quality indices of camels fed CON and SAL diets are shown in Table 5. Compared to milk from CON camels, milk from SAL camels contained lower (*p* < 0.05) values of UFA, MUFA, and OCFA. Furthermore, HFA and h/H values were decreased by the salt addition. On the other hand, the AI (*p* < 0.01), TI (*p* < 0.10), and HFA (*p* < 0.01) values were greater in SAL milk compared to the CON group.

### 3.3. Transfer Rates of Fatty Acids from Feed to Milk

The intake, milk output, and transfer rates of oleic, LA, and ALA acids are shown in Table 6. The transfer rate of oleic and ALA acids decreased with the SAL diet, whereas the transfer rate of LA was not affected by the dietary treatment.

### 3.4. Principal Component Analysis of Fatty Acid Composition of Camel Milk

**Figure 1:**
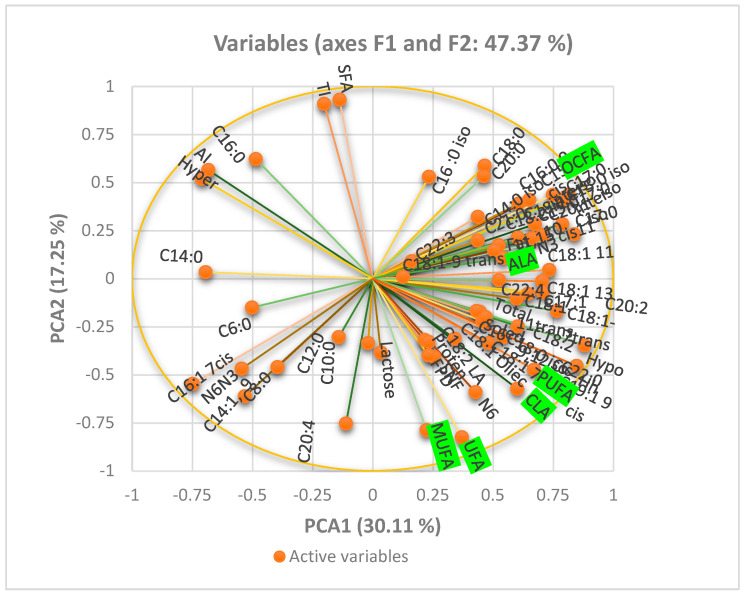
Principal component analysis of the fatty acid composition of milk produced from camels fed concentrates with 2 levels of salt: CON (1.4% salt; *n* = 12) and SAL (4.3% salt; *n* = 12).

The PCA (Figure 1) was performed to explore the relationships among the variables, with the first 2 principal components (PCA1 and PCA2) explaining a total of 47.37% of the variance. PCA1 accounted for 30.1% of the variance, whereas PCA2 explained 17.3%. The biplot revealed that variables such as MUFA, PUFA, and OCFA were strongly correlated and contributed positively to PCA1. However, variables such as C6:0 and C14:0 were negatively associated with PCA1 but had a strong influence on PCA2. The proximity of the vectors in the correlation circle highlights the positive correlations between FA subgroups, while the divergence of C6:0 and C14:0 suggests an inverse relationship with UFA.

### 3.5. Blood Metabolites

As shown in Table 7, salt supplementation increased blood insulin values (*p* < 0.05), without affecting blood glucose levels. Nevertheless, SAL camels had lower levels of total protein (*p* < 0.01), albumin (*p* < 0.10), and globulin (*p* < 0.01) compared to CON camels. No significant differences were detected in blood urea, the globulin-to-albumin ratio, or triglycerides between the two groups. We detected a positive correlation (r = 0.55; *p* < 0.01) between insulin levels in plasma and milk.

## 4. Discussion

### 4.1. Milk Production and Milk Insulin Levels

The effect of dietary salt on milk production in dairy camels is of significant interest due to its potential implications for camel husbandry and milk yield optimization. In the current study, the amount of salt provided by the concentrate mixture was 83 g/d for the CON group and 250 g/d for the SAL group, resulting in a difference of 167 g/d in consumed salt. To maintain the hydration and electrolyte balance, SAL camels consumed more water compared to the CON animals, as previously reported by Al Jassim [24].

Despite the difference in salt intake, no changes in milk yield or most milk components were observed between camels fed the CON or SAL concentrate mixtures. Since camels in the two treatment groups consumed the same amounts of concentrate (5.88 kg/d) and alfalfa hay (3.54 kg/d) on a DM basis, minimal impact on milk production was expected. Similarly, Onjoro et al. [25] found that common salt had an insignificant effect on milk yield in lactating camels [25]. In addition, increased dietary salt had no effect on milk production in dairy cows, despite inducing alterations in rumen fermentation [26].

In the literature, there is a wide variation in camel milk composition depending on factors like breed, diet, and environment. In this study, the milk component values fall within the range reported in a comprehensive study that evaluated the milk composition of 3 camel breeds (Majaheim, Wadah, and Hamra) in Saudi Arabia [27,28].

We hypothesized that moderate salt supplementation would affect blood insulin levels and potentially increase the insulin passing from blood to milk through putative leaky tight junctions in the mammary gland. Blood insulin levels increased in SAL animals, and we detected a positive correlation between blood and milk insulin levels. However, milk insulin levels did not vary significantly between treatments, although they were numerically 16% higher in SAL compared to CON camels. It seems that the integrity of mammary tight junctions was not affected by moderate salt supplementation in the present study, since milk lactose levels (which can be an indicator of tight junction permeability) did not vary between treatments. Milk insulin values observed in the present study are similar to levels previously reported in camel milk and are slightly higher than values reported in dairy cows (0.4 to 1.0 μg/L) [29,30].

### 4.2. Milk Fatty Acids

In the present study, the predominant milk FA were palmitic (28.0 ± 0.2%), oleic (C18:1 cis9; 19.7 ± 0.2%), stearic (13.5 ± 0.4%), myristic (10.9 ± 0.2%), and palmitoleic (C16:1 cis7; 9.3 ± 0.1%) acids, which accounted for 81% of the total FA. Additionally, SFA represented 59% of the total FA. These FA values are consistent with previous findings on camel milk as previously reviewed by Benmeziane-Derradji [7]. The concentration of caproic acid (C6:0) was 0.07 ± 0.01%, although, in other studies, C6:0 was not detected in camel milk [31]. Short-chain FA is thought to improve the gut microbiota environment and health [32]. Compared to the published values in dairy cows [33], camel milk in the present study contained higher levels of UFA (41% vs. 30–34%), MUFA (37% vs. 25–30%), and PUFA (4% vs. 3–5%). The CLA content did not vary between treatments and averaged 0.70 ± 0.02%. Based on the CLA content observed in this study, camel milk could meet human daily CLA requirements, which have been associated with a 43% reduction in non-fatal myocardial infarction risk from consuming 0.33 g of CLA per day [34].

Supplementation with salt reduced the levels of UFA and MUFA, which in turn negatively impacted lipid quality indices. This finding suggests that SAL treatment may reduce the proportion of health-promoting FA, as UFAs are known to have protective cardiovascular effects, including lowering LDL cholesterol and reducing inflammation [35]. Furthermore, OCFA, such as C15:0 and C17:0, which are associated with health benefits like reduced risks of type 2 diabetes and metabolic syndrome [36], were lower in the SAL group.

Although not significant (*p* = 0.16), SAL supplementation increased the n6/n3 ratio from 3.9 to 5.0 (+26%), suggesting a potential shift toward a more pro-inflammatory profile. Lower ratios (closer to 1:1) are generally associated with anti-inflammatory effects [37]. These overall changes in FA profile suggest that salt supplementation may reduce the health benefits of camel milk.

Both the atherogenicity and thrombogenicity indices increased or tended to increase with salt supplementation. The atherogenicity index reflects the balance of lipids that are prone to contributing to artery plaque formation [29], while the thrombogenicity index predicts the potential for blood clot formation, with higher values indicating a greater risk. Despite this, camel milk from both treatments had lower atherogenicity indices (1.63 to 1.91) than those reported in bovine dairy products such as milk, butter, and cheese (2.03) as reported by Ulbricht and Southgate [38].

Camels supplemented with SAL produced milk with lower concentrations of hypocholesterolemic FA (h) but greater levels of hypercholesterolemic (H) FA, resulting in a decreased h/H ratio. The H value is primarily influenced by SFA, especially lauric (C12:0), myristic (C14:0), and palmitic acids (C16:0) [38]. A lower h/H ratio is associated with an increased risk of cardiovascular diseases, as it reflects a higher proportion of FA that can raise LDL cholesterol [39].

Overall, SAL treatment led to significant changes in milk FA profile and lipid quality indices. The most concerning effects include reductions in MUFA and hypocholesterolemic FA and an increase in hypercholesterolemic FA. This leads to a shift towards a less favorable milk fat profile, which may increase the risk of cardiovascular diseases. The significant rise in the atherogenic index and decrease in the h/H ratio further support the negative impact of salt supplementation on the milk’s lipid profile.

The reason why SAL affected the milk FA profile without altering milk yield or milk fat content is not clear but could be related to increased rumen osmolarity, which can affect fermentation processes. Similar effects have been reported in goats [40] and beef cattle [7]. Increased water intake in SAL camels could be a response to higher rumen osmolarity, which may reduce bacterial adhesion to feed particles and increase the turnover rate of solid and liquid phases in the rumen [40].

The transfer rate of oleic and alpha-linolenic acids decreased by SAL treatment. These FA are typically derived from dietary sources or produced via desaturation in the mammary gland [41]. The lower transfer rate of C18:1 and C18:3, *n*-3 in SAL treatment suggests that dietary salt may alter rumen biohydrogenation as mentioned above.

Overall, milk from camels supplemented with a moderate amount of salt would have negative effects on human health by potentially increasing cholesterol, LDL, and triglycerides, decreasing high-density lipoprotein cholesterol [42], and increasing the likelihood of developing atherosclerosis [38].

### 4.3. Blood Metabolites

Blood insulin levels were greater in SAL camels compared to the CON animals. Wu et al. [43] reported that increased salt intake has been associated with alterations in insulin regulation due to osmotic balance changes and increased glucose utilization in tissues. Higher insulin levels could also be linked to increased nutrient absorption and metabolic demands imposed by the elevated osmolarity in the SAL group [33]. Additionally, Petrie et al. [44] reported that increased Na^+^ intake can lead to higher circulating insulin levels in response to glucose challenges, particularly in salt-sensitive individuals. This hyperinsulinemic response has been observed in both human and animal models, likely driven by Na^+^ effects on cellular ion channels and pancreatic function. Insulin promotes body lipogenesis [45], favoring the synthesis of SFA while reducing the mobilization of stored UFA, which could lead to lower UFA levels in milk [46], as we observed in the current study.

Blood glucose concentrations increased by 6% in SAL camels, but this increment was not significant (*p* = 0.31). Increased salt consumption induces insulin resistance [36], which might explain the mismatching between insulin and glucose values. The reduced total protein (*p* < 0.01), albumin (*p* < 0.10), and globulin (*p* < 0.01) in SAL camels may be attributed to a dilution effect due to increased water intake.

Although we did not observe a significant difference between treatments in milk insulin levels, a positive correlation between insulin in blood and milk was detected. Nevertheless, Zinicola and Bicalho [47] found no correlation between plasma insulin concentrations and insulin levels in colostrum. The transfer of insulin into milk is a complex process influenced by regulatory mechanisms inherent to the mammary gland, indicating that milk insulin may not directly reflect changes in systemic blood insulin levels.

## 5. Conclusions

This study provides valuable insights into the effects of dietary salt supplementation on dairy camels. While moderate salt inclusion did not negatively affect milk yield or major milk components, it altered the FA profile, reducing beneficial FA and increasing the atherogenic index. Although milk insulin levels remained unchanged, salt supplementation increased blood insulin levels without affecting blood glucose, suggesting a potential impact on the animal’s insulin sensitivity. Additionally, salt supplementation decreased total protein, albumin, and globulin levels, indicating potential effects on protein metabolism and fluid balance. These findings suggest that careful consideration should be given to dietary salt levels in camel feeding regimes, balancing the need to meet mineral requirements with potential impacts on milk quality and human health. Further research is warranted to fully elucidate the complex interplay between dietary salt and camel milk composition, particularly concerning insulin dynamics and FA metabolism.

## Figures and Tables

**Table 1:** Ingredients of the experimental control (CON) and high-salt (SAL) concentrates.

Ingredient, %	CON	SAL
Corn grain	76.0	76.0
Barley grain	15.4	15.4
Soybean meal 44%	2.0	2.0
Salt	1.4	4.3
Ca Carbonate	2.9	0.0
Molasses	1.6	1.6
Vitamin and mineral mixture	0.7	0.7

**Table 2:** Chemical composition and fatty acid profile of the alfalfa hay and the experimental CON and SAL concentrates (g/kg DM basis).

Item	Alfalfa Hay	CON	SAL
**Chemical composition, %**			
Dry matter	93.1	90.3	90.6
Crude protein	13.9	11.8	11.6
Ether extract	1.03	2.61	2.59
NDF	43.0	42.8	42.1
ADF	21.9	18.3	18.6
Ash	10.3	5.48	5.63
**Fatty Acid Profile, %**			
C12:0	0.81	-	0.21
C14:0	0.97	0.08	0.18
C15:0	0.59	-	-
C16:0	21.0	13.7	12.9
C16:1 cis9	1.34	-	-
C17:0	0.46	0.13	0.19
C18:0	4.19	2.41	2.57
C18:1 cis9 (OA)	3.68	30.6	31.9
C18:0 9 trans	0.28	0.78	0.72
C19:0	-	-	0.77
C18:2 cis9,12 (LA)	27.3	49.2	46.8
C18:2 cis6,7	0.24	-	1.16
C20:0	2.05	0.64	0.56
C18:3 cis9,12,15 (ALA)	34.3	2.04	1.67
C21:0	-	0.38	0.29
C22:0	2.16	-	-
C23:0	0.67	-	-

**Table 3:** Influence of dietary salt level on milk yield and milk composition in dairy camels (*n* = 12 for each treatment).

Item	Treatments ^1^		SEM	*p* Value <
	CON	SAL		
Water consumption, L/d	34.0	35.8	0.58	0.01
Milk yield ^2^	4.48	4.29	0.43	0.67
Milk fat, %	2.25	2.18	0.12	0.72
Milk protein, %	2.33	2.40	0.08	0.54
Milk lactose, %	4.49	4.46	0.10	0.75
Milk total solids, %	9.66	9.62	0.25	0.92
Milk solid non-fat, %	7.45	7.41	0.17	0.87
Milk freezing point depression	0.57	0.55	0.01	0.27
Milk insulin, μg/L	1.19	1.38	0.51	0.57

^1^ Concentrates with 2 levels of salt: CON (1.4% salt) and SAL (4.3% salt). ^2^ Calves were separated from their mothers at 5 a.m., and milking was done at 9 a.m. Therefore, milk yield does not include the amount of milk consumed by the calves during the night (from after the p.m. milking until 5 a.m.).

**Table 4:** Influence of dietary salt level on milk fatty acid profile (g/100 g FA) in dairy camels (*n* = 12 for each treatment).

Fatty Acid	Treatments ^1^		SEM	*p* Value <
	CON	SAL		
C6:0	0.06	0.09	0.01	0.03
C8:0	0.09	0.12	0.02	0.04
C10:0	0.12	0.15	0.01	0.29
C12:0	0.79	0.81	0.02	0.98
C13:0	0.07	0.08	0.00	0.89
C14:0 iso	0.22	0.16	0.01	0.18
C14:0	10.29	11.45	0.24	0.03
C14:1, cis9	1.02	1.11	0.02	0.67
C15:0	1.17	1.03	0.03	0.22
C15:0 iso	0.34	0.28	0.01	0.04
C15:0 anteiso	0.78	0.66	0.04	0.05
C16:0	26.6	29.4	0.14	0.01
C16:0 iso	0.79	0.46	0.05	0.02
C16:1 cis7	8.88	9.76	0.03	0.33
C16:1 cis9	0.49	0.41	0.04	0.09
C17:0	0.73	0.63	0.04	0.18
C17:0 iso	0.48	0.44	0.01	0.30
C17:0 anteiso	0.88	0.73	0.01	0.01
C17:1 cis10	0.57	0.48	0.01	0.02
C18:0	13.8	13.1	0.41	0.25
C18:0 iso	0.20	0.12	0.00	0.86
C18:1 trans7	0.99	0.76	0.06	0.04
C18:1 trans9	1.20	1.37	0.15	0.63
C18:1 trans11	1.87	1.00	0.01	0.05
C18:1 cis9	20.6	18.8	0.22	0.01
C18:1 cis11	1.52	1.40	0.08	0.36
C18:1 cis13	0.34	0.32	0.03	0.84
C18:1 cis14	0.48	0.43	0.02	0.18
C18:1 cis7	0.22	0.15	0.00	0.81
C18:2 trans11,15	0.32	0.34	0.06	0.56
C18:2 trans9,12	0.23	0.14	0.00	0.03
C18:2 cis 9,12 (LA) ^2^	2.49	2.66	0.10	0.20
C18:2 cis9 trans11(CLA) ^3^	0.76	0.63	0.02	0.17
C18:3 cis9,12,15 (ALA) ^4^	0.44	0.37	0.01	0.22
C19:1 cis9	0.14	0.08	0.00	0.89
C20:0	0.34	0.32	0.01	0.51
C20:1 11 cis	0.17	0.17	0.13	0.92
C20:2 cis11,14	0.05	0.04	0.00	0.28
C20:4 cis5,8,11,14	0.18	0.24	0.04	0.31
C21:0	0.06	0.06	0.01	1.00
C22:0	0.08	0.07	0.00	0.61
C22:3	0.08	0.05	0.01	0.33
C22:4 cis7,10,13,16	0.12	0.11	0.01	0.44

^1^ Concentrates with 2 levels of salt: CON (1.4% salt) and SAL (4.3% salt). ^2^ LA: linoleic acid. ^3^ CLA: conjugated linolenic acid. ^4^ ALA: alpha-linolenic acid.

**Table 5:** Influence of dietary salt level on total milk fatty acid profile (g/100 g FA) and lipid quality indices in dairy camels (*n* = 12 for each treatment).

Item	Treatments ^1^		SEM	*p* Value <
	CON	SAL		
Fatty acid group ^2^				
SFA	57.7	59.5	0.02	0.16
UFA	42.3	40.0	0.27	0.04
MUFA	37.9	35.8	0.21	0.03
PUFA	4.41	4.20	0.06	0.51
OCFA	5.12	4.37	0.24	0.04
* n*-3	0.77	0.66	0.04	0.26
* n*-6	2.87	2.91	0.07	0.85
n6/n3	3.94	4.97	0.04	0.16
Lipid quality indices				
Atherogenic index (AI)	1.63	1.91	0.01	0.01
Thrombogenic index (TI)	1.91	2.06	0.01	0.10
Hypocholesterolemic FA	31.1	28.1	0.20	0.01
Hypercholesterolemic FA	37.7	41.6	0.41	0.01
h/H ^3^	0.83	0.68	0.01	0.01

^1^ Concentrates with 2 levels of salt: CON (1.4% salt) and SAL (4.3% salt). ^2^ SFA: saturated fatty acid; UFA: unsaturated fatty acid; MUFA: monounsaturated fatty acid; PUFA: polyunsaturated fatty acid; OCFA: odd chain fatty acid; n3: omega 3; n6: omega 6. ^3^ h/H: hypocholesterolemic/hypercholesterolemic FA.

**Table 6:** Influence of dietary salt level on the transfer rate of oleic, linoleic, and alpha-linolenic FA from feed into the milk of dairy camels (*n* = 12 for each treatment).

Item	Treatments ^1^		SEM	*p* Value <
	CON	SAL		
** Oleic acid**				
Intake (g/d)	0.33	0.34	0.01	0.03
Output (g/d)	0.70	0.60	0.02	0.01
Transfer rate (%)	213	177	5.08	0.02
** Linoleic acid**				
Intake (g/d)	0.31	0.32	0.01	0.79
Output (g/d)	0.08	0.09	0.01	0.88
Transfer rate (%)	26.4	26.6	1.21	0.88
** alpha-Linolenic acid**				
Intake (g/d)	0.32	0.34	0.01	0.001
Output (g/d)	0.02	0.01	0.01	0.104
Transfer rate (%)	4.66	3.53	0.50	0.003

^1^ Concentrates with 2 levels of salt: CON (1.4% salt) and SAL (4.3% salt).

**Table 7:** Influence of dietary salt level on blood metabolites diary camels (*n* = 12 for each treatment).

Item	Treatments ^1^		SEM	*p* Value <
	CON	SAL		
Insulin, μg/L	0.22	0.31	0.03	0.05
Glucose, mg/dL	51.1	54.0	1.90	0.31
Total protein, mg/dL	42.8	35.9	1.53	0.01
Urea, mg/dL	29.9	27.2	1.12	0.13
Albumin, g/dL	4.33	3.67	0.23	0.07
Globulin, g/dL	38.5	32.2	1.49	0.01
G/A	0.112	0.114	0.001	0.11
Triglycerides, mg/dL	35.1	37.1	2.09	0.34

^1^ Concentrates with 2 levels of salt: CON (1.4% salt) and SAL (4.3% salt).

## Data Availability

The original contributions presented in this study are included in the article. Further inquiries can be directed to the corresponding author.
